# Effectiveness of olanzapine for hyperemesis gravidarum in individuals without a psychiatric history: a case report

**DOI:** 10.1186/s12884-026-08893-w

**Published:** 2026-03-03

**Authors:** Hiromitsu Kaneko, Rika Tanimura, Tsuneaki Kenzaka, Noriko Seki, Yasushi Mizutani

**Affiliations:** 1 The Center for Clinical Training, Japanese Red Cross Society Himeji Hospital, Himeji, Japan; 2 Department of Obstetrics and Gynecology, Japanese Red Cross Society Himeji Hospital, Himeji, Japan; 3 Department of Internal Medicine, Hyogo Prefectural Tamba Medical Center, Tamba, Japan; 4 Division of Community Medicine and Career Development, Kobe University Graduate School of Medicine, Kobe, Japan

**Keywords:** Pregnancy, hyperemesis gravidarum, olanzapine, treatment, nausea and vomiting in pregnancy, refractory

## Abstract

**Background:**

Hyperemesis gravidarum (HG) is characterized by severe nausea and vomiting during pregnancy, and can markedly impair quality of life as well as cause maternal and fetal complications. Although a stepwise treatment strategy with antiemetics and nutritional support is commonly used, some cases remain refractory. Olanzapine, an atypical antipsychotic, has shown potential benefit in HG, although most reports involve patients with psychiatric comorbidities. We describe a patient with persistent HG in late gestation without psychiatric history, in whom adjunctive olanzapine was temporally associated with improvement in nausea and vomiting symptoms.

**Case presentation:**

A 42-year-old woman (Gravida 2, Para 0) conceived through frozen-thawed embryo transfer and was diagnosed with HG at gestational week 8. Despite intravenous fluids and metoclopramide, symptoms persisted, and she was hospitalized at 32 weeks due to poor maternal weight gain and fetal growth restriction. On admission, her nausea was severe (Numerical Rating Scale [NRS]: 10), with a Pregnancy-Unique Quantification of Emesis and Nausea (PUQE-24) score of 15 and persistent vomiting up to 18 episodes/day. Despite no prior psychiatric history, she developed depressive symptoms during pregnancy. Olanzapine was started at 2.5 mg/day and titrated to 10 mg/day by 35 weeks; total parenteral nutrition was initiated concurrently. By 35 weeks, her nausea improved (NRS: 10/10 to 3.5/10; PUQE-24: 15 to 9) and vomiting decreased to 9 episodes/day. A cesarean section was performed at 35 weeks and 2 days owing to acute pulmonary edema. A 2,160-g female infant was delivered with Apgar scores of 2, 4, and 5. The neonate was admitted to the neonatal intensive care unit due to transient respiratory distress (ventilation: 2 days; oxygen: 10 days), with a normal brain MRI prior to discharge and no evidence of hypoxic–ischemic encephalopathy. Maternal gastrointestinal symptoms resolved immediately after delivery.

**Conclusions:**

This case highlights the potential role of olanzapine in refractory HG management, even in the absence of pre-existing psychiatric comorbidities. It also underscores the importance of early nutritional intervention and multidisciplinary management, particularly when prolonged symptoms contribute to declining quality of life and depressive affect. Further case reports are required to confirm this therapeutic effect of olanzapine.

## Background

Nausea and vomiting of pregnancy (NVP) affects approximately 50–80% of pregnant women [1]. It typically begins around gestational weeks 5–6, peaks at weeks 9–10, and resolves spontaneously by weeks 12–16 in most cases [2]. However, 0.3–3% of women develop hyperemesis gravidarum (HG), a more severe condition characterized by significant weight loss (more than 5% of pre-pregnancy body weight), ketonuria, dehydration, and electrolyte imbalance, often requiring hospitalization [3]. Symptoms persisting beyond 22 weeks occur in up to 10% of cases, and a small proportion of patients continue to experience symptoms until delivery [4]. Because persistent nausea and vomiting in late gestation may overlap with pregnancy-specific disorders and non-obstetric conditions, HG remains a diagnosis of exclusion and warrants careful evaluation for alternative etiologies. HG is also associated with psychological morbidity, including depression and anxiety symptoms. A systematic review reported significantly higher depression and anxiety symptom scores among women with HG compared with controls without notable NVP, underscoring the importance of psychological care and support as part of comprehensive management [5]. HG substantially impairs maternal quality of life (QOL), with marked deterioration in health-related QOL documented using both generic instruments and NVP/HG-specific measures such as the NVPQOL questionnaire [6–8]. HG may result in complications such as Wernicke encephalopathy and deep vein thrombosis.

It also increases the risk of adverse fetal outcomes, including fetal growth restriction and preterm birth. Therefore, timely and appropriate medical intervention is critical [1, 9].

The pathophysiology of NVP and HG remains incompletely understood but is considered multifactorial [10, 11]. Contributing factors include hormonal influences such as elevated human chorionic gonadotropin levels, transient hyperthyroidism, and maternal sensitivity to fetal–placental growth differentiation factor, along with genetic predisposition, gastrointestinal changes, and psychological factors, including depression and anxiety.

According to algorithms from professional societies [1, 12] and systematic reviews [13], management typically follows a stepwise approach. It begins with lifestyle modifications such as small, frequent meals and rest. It then progresses to pharmacologic interventions, including vitamin B6 supplementation and antiemetics from different pharmacologic classes, such as pyridoxine with or without doxylamine, antihistamines, dopamine antagonists, and serotonin (5-HT3) antagonists. When necessary, it advances to intravenous hydration and nutritional support. Because HG is a diagnosis of exclusion, improvement with standard stepwise management supports a less severe NVP phenotype; conversely, the term HG is often reserved for cases that require escalation of care due to persistent or severe symptoms. Nevertheless, some patients remain unresponsive to conventional therapies. In refractory HG, prolonged symptoms may precipitate a decline in QOL and the emergence of depressive affect. For refractory cases, additional options—although not uniformly recommended across guidelines—may include corticosteroids, *Helicobacter pylori* eradication therapy, and selected psychotropic agents (e.g., mirtazapine or benzodiazepines) in carefully selected patients. Given that olanzapine is an antagonist of emesis-related receptors (e.g., D_2_/5-HT2/5-HT3/H1) and it has psychotropic properties, it may serve as a dual-acting therapeutic option, targeting both emesis and depressive symptoms—even in the absence of pre-existing psychiatric comorbidities.

Although HG is not very common, its maternal–fetal burden is substantial and is frequently accompanied by depressive and anxiety symptoms [14], which can further erode QOL and treatment adherence. While olanzapine is primarily indicated for psychiatric disorders (e.g., schizophrenia and bipolar disorder), the literature on its use for HG has largely involved women treated for psychiatric comorbidity, with improvement in HG described as a secondary observation. Overall, the available evidence for the use of olanzapine to treat HG remains limited, and is largely confined to case reports and small case series. In addition to its psychiatric indications, olanzapine is also used as an antiemetic for chemotherapy-induced nausea and vomiting; however, indications for HG have not been established. Together with available pharmacovigilance and review data suggesting no consistent teratogenic signal with low-dose olanzapine [15], these considerations prompted us to explore a clinically important evidence gap through this case report.

Herein, we report a case of treatment-resistant HG in a patient without a psychiatric history, in whom symptom burden was ameliorated during initiation and dose escalation of adjunctive olanzapine in combination with nutritional support. Evidence of clinical efficacy is required in additional cases before olanzapine can be considered a late-line adjunct for refractory HG without pre-existing psychiatric comorbidity, particularly when prolonged symptoms are accompanied by depressive affect and declining QOL. Multidisciplinary care and early nutritional support should accompany use, along with metabolic monitoring to mitigate potential adverse effects.

## Case presentation

A 42-year-old woman (Gravida 2, Para 0) with a height of 151 cm and a pre-pregnancy weight of 60 kg conceived via frozen-thawed embryo transfer. She was hospitalized at another institution for HG from 8 to 14 weeks of gestation and received intravenous fluids and metoclopramide. After discharge from the referring hospital, during outpatient follow-up, her body weight increased from 54.9 kg at 15 weeks to 61.0 kg by 28 weeks, then plateaued and slightly decreased. At 31 weeks, she demonstrated inadequate maternal weight gain (60.4 kg) and fetal growth restriction, with an estimated fetal weight (EFW) of 1238 g (− 2.1 standard deviation [SD]). She was referred and admitted to our institution at 32 weeks and 1 day. Her medical history included uterine fibroids without glucose intolerance. She reported no family history of HG and worked as a nurse. The uterine fibroid had been diagnosed at the referring hospital and was asymptomatic; in accordance with patient preference, management comprised infertility treatment rather than fibroid-directed therapy. Nausea severity was assessed using both the Numerical Rating Scale (NRS) as a commonly used self-reported measure [16] and the 24-hour Pregnancy-Unique Quantification of Emesis and Nausea (PUQE-24) score as a standardized measure [17]. NRS was assessed using an 11-point NRS (0 = no nausea, 10 = worst imaginable nausea) reported by the patient. The PUQE-24 score is calculated based on vomiting episodes, duration of nausea, and episodes of retching/dry heaves. A PUQE-24 score ≥ 13 indicates severe symptoms, 7–12 moderate symptoms, and ≤ 6 mild symptoms. The PUQE-24 has been shown to correlate strongly with QOL during pregnancy [8, 17]. At admission, although she was receiving metoclopramide for nausea, her nausea severity was rated 10 on the NRS. The PUQE-24 score was 15 (severe). Although she reported no prior psychiatric history, depressive affect was clinically noted during admission. Physical examination revealed no abdominal distension or tenderness, and fetal movement was reassuring. Ultrasonography showed an EFW of 1,261 g (− 2.1 SD), transverse lie, dolichocephaly, marginal placenta previa (posterior right wall), and a 10 cm intramural fibroid on the posterior left uterine wall. A non-stress test showed no uterine contractions and a reassuring fetal heart rate pattern (category I). Laboratory testing indicated microcytic anemia and malnutrition, reflected by hypoalbuminemia, suggesting poor nutritional status (Table 1). Mild leukocytosis and a slight CRP elevation were present without clinical features of acute infection. Urinalysis was negative for ketone bodies, likely reflecting prior fluid resuscitation.

**Table 1:** Selected laboratory findings on admission—hematology, biochemistry, and renal and endocrine profile

Parameter	Result	Reference range
White blood cell count	8700/µL	3300–8600 /µL
Hemoglobin, blood	9.9 g/dL	11.5–15 g/dL
Mean corpuscular volume	78.4 fL	83–99 fL
Platelet count	302 10^3^/µL	150–350 10^3^/µL
C-reactive protein	0.23 mg/dL	0–0.14 mg/dL
Albumin	3.3 g/dL	4.1–5.1 g/dL
Sodium	137 mmol/L	138–145 mmol/L
Potassium	4.2 mmol/L	3.6–4.8 mmol/L
Chloride	107 mmol/L	101–108 mmol/L
Blood urea nitrogen	9.3 mg/dL	8–20 mg/dL
Creatinine	0.63 mg/dL	0.46–0.79 mg/dL
Blood glucose	82 mg/dL	70–109 mg/dL
Aspartate aminotransferase	10 U/L	13–30 U/L
Alanine aminotransferase	7 U/L	7–23 U/L
Total bilirubin	0.42 mg/dL	0.4–1.5 mg/dL
Thyroid-stimulating hormone	4.610 mIU/L	0.61–4.23 mIU/L
Free thyroxine	0.846 pg/mL	0.9–1.7 pg/mL

**Fig. 1:**
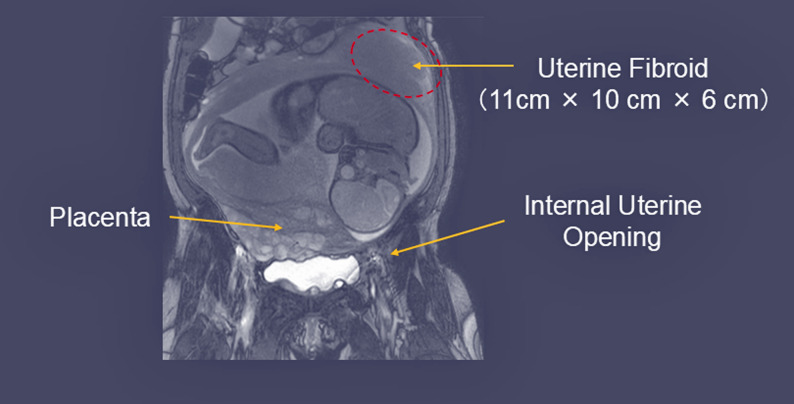
MRI T2-weighted image of the uterus at 34 weeks and 3 days of gestation. MRI, magnetic resonance imaging

Given the persistence of nausea and vomiting into the third trimester, we performed a targeted evaluation.　Since HG is a diagnosis of exclusion, we assessed alternative etiologies, including pregnancy-specific disorders (e.g., preeclampsia with severe features/HELLP syndrome) as well as neurological, endocrine/metabolic, gastrointestinal, infectious, medication toxicity-related, vestibular, and psychiatric causes. She had no clinical features suggestive of acute infection or medication toxicity (e.g., fever, diarrhea, or recent exposure to emetogenic agents). She remained normotensive on admission. Brain magnetic resonance imaging (MRI) revealed no intracranial abnormalities. Laboratory results on admission showed no significant electrolyte disturbance or hyperglycemia, and liver enzyme levels were not elevated (Table 1). Platelet count and bilirubin levels were within the normal range. Thyroid function tests showed mildly elevated TSH (4.6 mIU/L) with borderline low free T4 (0.84 pg/mL). However, repeat testing 2 weeks later demonstrated spontaneous normalization (TSH: 2.4 mIU/L, free T4 0.94 pg/mL) without thyroid-specific treatment. Upper gastrointestinal endoscopy excluded malignancy or structural lesions, and fecal occult blood testing was negative. MRI confirmed a 10-cm intramural fibroid on the posterior uterine wall (Fig. 1); therefore, mechanical compression by the enlarged uterus and fibroid could not be completely excluded as a contributing factor.

Following admission, the Nutrition Support Team (NST) implemented interventions including modification of dietary form, fluid therapy, and provision of high-calorie supplements such as nutritional drinks, jellies, and cheese. Depressive symptoms were clinically noted amid her prolonged illness and decline in QOL. Symptoms were not quantified using a validated scale (e.g., EPDS/PHQ-9). A psychiatric consultation was discussed but not pursued, and a formal psychiatric diagnosis was not established.

**Fig. 2:**
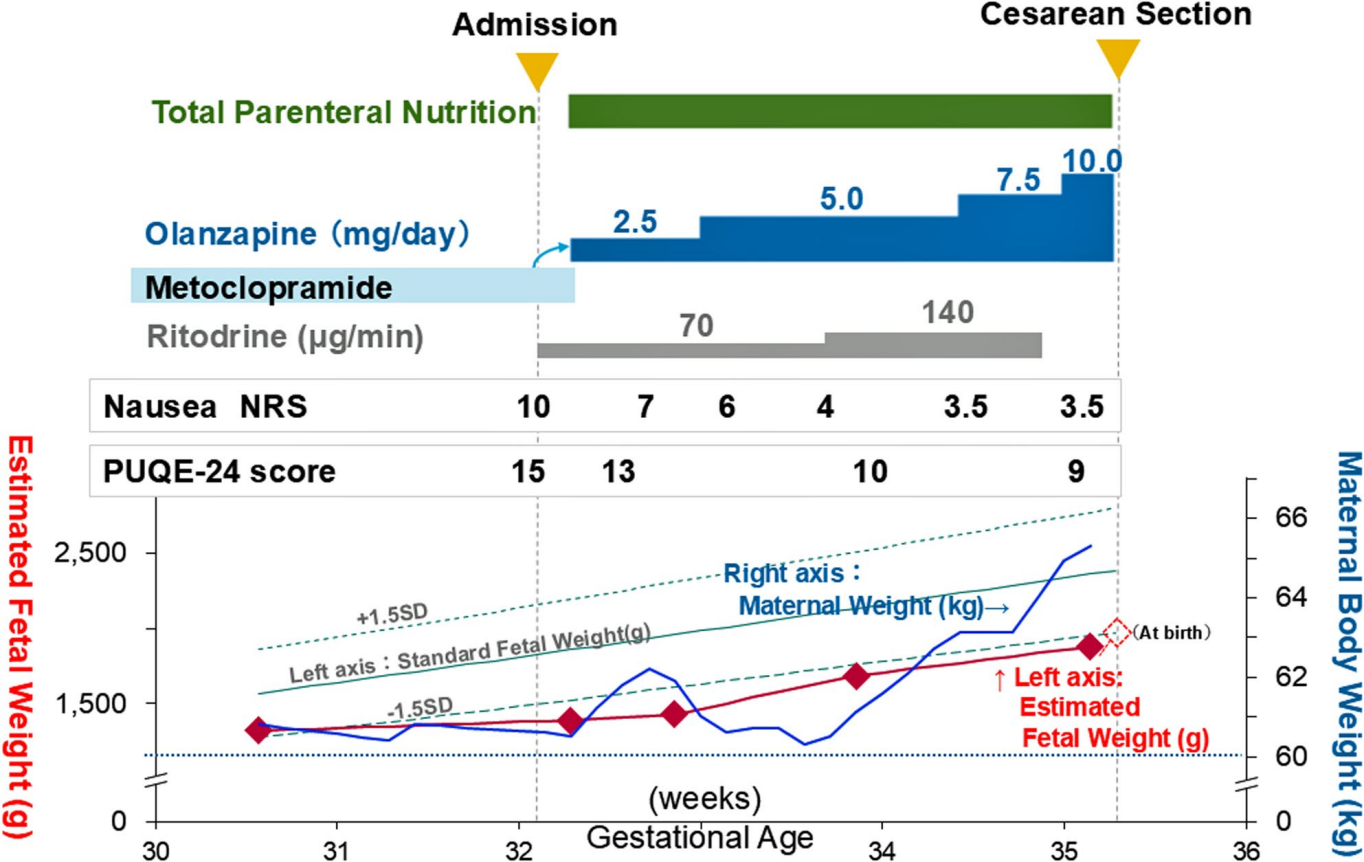
Clinical course following hospital admission. Nausea was assessed using two complementary patient-reported measures: The Numerical Rating Scale (NRS) to capture subjective nausea intensity (0 = no nausea, 10 = worst imaginable nausea) and the 24-hour Pregnancy-Unique Quantification of Emesis and Nausea score (PUQE-24), a standardized composite symptom-burden score (range 3–15; higher scores indicate more severe symptoms; severe is typically ≥ 13). SD, standard deviation

The use of metoclopramide failed to control emesis, and the QOL of the patient had deteriorated markedly in the absence of a psychiatric diagnosis. Following a risk–benefit discussion and shared decision-making, olanzapine was selected and initiated on hospital day 3 as a late-line adjunct to address refractory emesis accompanied by depressive affect (2.5 mg/day at 32 weeks). Because oral intake remained inadequate—the patient was consuming approximately 20% of hospital-provided meals with frequent vomiting—total parenteral nutrition (TPN) was also initiated at 32 weeks and 3 days (hospital day 3), and its prescription was not escalated thereafter. Symptom improvement was observed during the admission course: Nausea severity decreased from 10 to 3.5 on the NRS, and the PUQE-24 score decreased from 15 to 9 (Fig. 2). During olanzapine dose escalation to 10 mg/day by 35 weeks, postprandial nausea duration shortened to approximately 3 h, and vomiting episodes decreased from 18 to 9 per day, although vomiting did not fully resolve. Both maternal and fetal weight demonstrated upward trends toward 35 weeks.

Ritodrine was administered for uterine irritability from hospital day 1 until the day before cesarean delivery. Although an elective cesarean section was planned at 36 weeks and 4 days owing to marginal placenta previa, an emergency cesarean was performed at 35 weeks and 2 days for acute pulmonary edema (maternal indication). Final diagnoses were preterm labor at 35 weeks and 2 days, acute pulmonary edema, and marginal placenta previa. Estimated blood loss, including amniotic fluid, was 1390 mL. A female infant weighing 2,160 g was delivered, with Apgar scores of 2, 4, and 5. The neonate was admitted to the NICU due to transient respiratory distress, requiring mechanical ventilation for 2 days and supplemental oxygen for 10 days. No evidence of hypoxic-ischemic encephalopathy was observed, and brain MRI findings were normal prior to discharge. The infant was discharged on postnatal day 36 (corrected gestational age: 40 weeks and 3 days), and pediatric follow-up at 3 and 12 months (corrected age) was reassuring. Postoperatively, maternal nausea and vomiting resolved immediately, and her condition improved rapidly.

## Discussion

Management of NVP/HG is typically stepwise, escalating from dietary/lifestyle measures to antiemetics and, when needed, enteral or parenteral nutritional support [13]. However, a subset of patients remains refractory to treatment. Although no consensus on the definition of “refractory” HG has been established, the term is commonly used for persistent symptoms despite guideline-recommended therapy and adequate hydration/nutritional support [1, 2, 12]., A limitation in this case is that multiple antiemetics were not trialed before initiation of olanzapine; however, despite standard therapy that included metoclopramide, symptoms persisted into late gestation and required hospitalization and parenteral nutritional support. From this perspective, we consider this presentation to be consistent with HG. In such cases, alternative strategies—including treatment with atypical antipsychotics such as olanzapine and mirtazapine—have been explored. This case suggests that for patients with severe HG who also exhibit depressive affect, olanzapine may represent a suitable option by virtue of its dual antiemetic and psychotropic actions, even in the absence of pre-existing psychiatric comorbidities. We describe a case of refractory HG in a patient without a psychiatric history, in whom nausea severity and duration decreased during initiation and up-titration of olanzapine. Depressive symptoms were clinically observed during pregnancy.

Because symptom persistence into late gestation is uncommon, we reassessed the differential diagnosis and found no evidence of an alternative neurologic, endocrine/metabolic, gastrointestinal, infectious, medication toxicity-related, or psychiatric etiology, although mechanical compression by the enlarged uterus and fibroid could not be fully excluded. The immediate resolution of symptoms after delivery, in our case, further supports a pregnancy-related etiology.

This stepwise framework mirrored our patient’s course: at the referring hospital, she received metoclopramide and intravenous hydration, yet by 32 weeks symptoms persisted with inadequate maternal weight gain (60.4 kg) and fetal growth restriction (estimated fetal weight 1,238 g, -2.1 SD), prompting referral. Standard care begins with lifestyle modifications, such as initial pharmacologic interventions, including the use of vitamin B6 (pyridoxine) and ginger, while nonpharmacologic options, such as acupressure at the P6 point, may provide symptom relief. For moderate-to-severe cases, antiemetics are introduced. Metoclopramide, a dopamine and serotonin receptor antagonist, enhances gastric motility but may cause side effects such as drowsiness and dystonia. Ondansetron, a 5-HT3 antagonist, is widely used but carries potential safety concerns, including a possible association with congenital malformations. Promethazine, an antihistamine, is another therapeutic option. In severe HG, characterized by dehydration and significant maternal weight loss, intravenous fluids and thiamine supplementation are essential. Corticosteroids may be considered in resistant cases, though their safety profile in pregnancy remains debated. For truly refractory HG, agents such as olanzapine and mirtazapine have shown promise in recent case reports [18]. In our case, following admission, we promptly implemented NST-guided nutrition support including high-calorie oral supplements and TPN. Notably, a similar stepwise approach had been attempted at the referring institution; however, the absence of escalation to formal nutrition support likely contributed to persistent symptoms before referral. Following shared decision-making and a risk–benefit discussion, olanzapine was administered as a late-line adjunct to address refractory emesis accompanied by depressive affect. Subsequently, symptoms improved (NRS decreased and vomiting frequency halved). However, because initiation of TPN and initiation/up-titration of olanzapine occurred concurrently, the observed clinical improvement is likely to be multifactorial, and the specific contribution of olanzapine cannot be determined from this single case. In our case, the TPN prescription was maintained without escalation during the titration period, whereas nausea severity and duration were ameliorated in parallel with olanzapine dose escalation, suggesting a possible contribution of olanzapine to symptom relief.

Taking the pharmacological profile of olanzapine into consideration, its efficacy in managing HG may not necessarily depend on the presence of a psychiatric diagnosis. Olanzapine is an atypical antipsychotic of the thiobenzodiazepine class that antagonizes multiple neurotransmitter receptors, including dopaminergic (D_1_, D_2_, D_3_, and D_4_), serotonergic (5-HT_2_A, 5-HT2C, 5-HT3,and 5-HT6), adrenergic (α_1_), muscarinic, and histaminergic (H1) receptors [19]. This antagonism against multiple receptors may result in a broader antiemetic effect; however, it may also contribute to a diverse adverse-effect profile, including sedation/somnolence, orthostatic hypotension, anticholinergic effects (e.g., dry mouth and constipation), or extrapyramidal symptoms [20, 21]. Olanzapine is also associated with metabolic adverse effects such as weight gain/increased appetite and dysglycemia/dyslipidemia [20, 21]. Weight gain has been suggested to correlate with H1 receptor antagonism (and possibly with α_1_A, 5-HT2C, and 5-HT6 receptor activity) [22]. Because use during pregnancy has been associated with an increased risk of gestational diabetes and large-for-gestational-age (LGA) infants, monitoring of maternal weight and glucose during treatment is warranted [23]. While metoclopramide primarily targets D_2_ receptors and ondansetron selectively antagonizes 5-HT3 receptors, the broader receptor activity of olanzapine, particularly at D_2_, 5-HT2C, and 5-HT3 (which are implicated in nausea and emesis), has prompted its use in cases of nausea and vomiting refractory to standard antiemetics. Furthermore, because poor oral intake and weight loss are major clinical problems in HG, the appetite-stimulating effect of olanzapine (including secondary effects mediated by relief of nausea and vomiting) may facilitate recovery of oral intake, with appetite improvement after initiation having been reported in severe cases of HG [24].

To further contextualize our case, we conducted a literature review on olanzapine for HG. We searched PubMed (1946–June 30, 2025) using the terms (olanzapine OR zyprexa) AND (“hyperemesis gravidarum” OR “nausea and vomiting pregnancy” OR “morning sickness“). Reports not meeting criteria for HG or involving atypical contexts (e.g., substance use disorders) were excluded. In addition to the cases included in prior reviews, we identified a Japanese-language case report published in 2019 [24].

In total, six publications reported the use of olanzapine for HG [18, 24–28], including two review articles. Among these, we identified nine individual cases across four publications in which olanzapine was used to treat HG (six cases reported by Sharma et al. [26]; one by Galletta et al. [18], one by Sharma et al. [28]; and one by Kanemori et al. [24]). Only two cases lacked a documented psychiatric history (one reported by Galletta et al. [18] and one by Kanemori et al. [23]). The remaining seven cases were reported to have a history of bipolar II disorder or major depressive disorder. Most cases, however, involved patients with psychiatric disorders or mood disturbances for which olanzapine was primarily prescribed; improvement in HG was generally described as a secondary finding. Of note, a systematic review published in 2022 [18] (searching PubMed/MEDLINE through June 2022 without language restrictions) identified 2 olanzapine case reports for refractory HG; in both, olanzapine was prescribed primarily for psychiatric symptoms, with improvement in HG as a secondary outcome. Our updated review incorporates more recent publications from 2022 onward, as well as Japanese literature, and our case adds an additional example of adjunctive olanzapine use in late gestation, in a patient without a documented psychiatric history, alongside early NST-guided nutrition. To the best of our knowledge, prior to our report, only two HG cases treated with olanzapine have been described in patients without a documented psychiatric history.

Based on this case and prior reports [18, 24, 27], early nutritional intervention appears essential for patients with HG unresponsive to intravenous fluids and dopamine antagonists. In this case, earlier initiation of nutritional support, compared with the referring institution, where management focused on metoclopramide and intravenous hydration without escalation to formal nutrition support, may have contributed to more favorable outcomes. Recognition that olanzapine may alleviate HG symptoms could expand therapeutic options and improve QOL. Recent large-scale registries and case series have not demonstrated significant increases in teratogenic risk or adverse neonatal outcomes with low-dose olanzapine exposure during pregnancy [20]. Although monitoring for metabolic effects such as maternal weight gain and glucose intolerance is warranted, olanzapine may represent a practical and relatively safe treatment agent for refractory HG. Currently, olanzapine is primarily used as an antipsychotic and mood-stabilizing agent for psychiatric indications (e.g., schizophrenia and bipolar spectrum disorders). Consequently, reports in which olanzapine is used specifically to treat HG remain extremely limited. Accumulating further clinical experience and publishing additional case reports, including among patients without psychiatric comorbidities, will be crucial to clarify its therapeutic role.

## Limitations

This report has certain limitations. First, depressive affect was clinically observed during hospitalization, but it was not quantified using a validated instrument (e.g., the Edinburgh Postnatal Depression Scale or PHQ-9). Although a psychiatric consultation was offered, it was not pursued; therefore, no formal psychiatric diagnosis was established. Second, we did not use an NVP-specific quality-of-life measure (e.g., NVPQOL); accordingly, changes in QOL were inferred rather than directly measured. Nevertheless, the PUQE-24 has been reported to correlate strongly with NVPQOL [8], and the observed decrease in PUQE-24 scores is consistent with reduced symptom burden. Third, prior to initiation of olanzapine, escalation through multiple conventional antiemetics from different classes was incomplete; thus, we cannot exclude the possibility that other standard agents might have been effective. Fourth, a transient thyroid function abnormality (mildly elevated TSH with borderline low free T4) was observed on admission. It normalized spontaneously within 2 weeks without thyroid-specific treatment. Thyroid autoantibodies were not assessed, which limits our ability to fully exclude underlying autoimmune thyroid disease. Finally, because initiation of TPN and initiation/up-titration of olanzapine occurred in close temporal proximity, clinical improvement was likely multifactorial, and the specific contribution of olanzapine cannot be determined from this single case.

## Conclusion

We reported a patient with treatment-resistant HG in whom depressive affect was clinically observed during pregnancy despite the absence of a psychiatric history. Initiation and up-titration of olanzapine was followed by improvement in symptom burden (NRS and PUQE-24) and maternal weight trajectory. For HG cases unresponsive to treatment with intravenous fluids and dopamine antagonists, early involvement of an NST is essential. The use of olanzapine may be beneficial for HG refractory to standard therapy, particularly when prolonged symptoms contribute to declining QOL with depressive affect. As a single-case report without standardized mood assessments and with concomitant nutritional interventions (including TPN), our findings do warrant cautious interpretation; prospective case series using validated mood scales and predefined nutrition algorithms could better delineate olanzapine’s psychotropic versus antiemetic contributions.

## Data Availability

No datasets were generated or analysed during the current study.

## Abbreviations

HG: Hyperemesis gravidarum
NVP: Nausea and vomiting of pregnancy
NRS: Numerical rating scale
NST: Nutrition support team
QOL: Quality of life
EFW: Estimated fetal weight
MRI: Magnetic resonance imaging
SD: Standard deviation
PUQE-24: 24-hour pregnancy-unique quantification of emesis and nausea
